# Gross and histopathological evaluation of human inflicted bruises in Danish slaughter pigs

**DOI:** 10.1186/s12917-016-0869-3

**Published:** 2016-11-08

**Authors:** Kristiane Barington, Jens Frederik Gramstrup Agger, Søren Saxmose Nielsen, Kristine Dich-Jørgensen, Henrik Elvang Jensen

**Affiliations:** 1 Department of Veterinary Disease Biology, Faculty of Health and Medical Sciences, University of Copenhagen, Ridebanevej 3, DK-1870 Frederiksberg C, Denmark; 2 Department of Large Animal Sciences, Faculty of Health and Medical Sciences, University of Copenhagen, Frederiksberg C, Denmark

**Keywords:** Age assessment, Bruise, Forensic, Pig

## Abstract

**Background:**

Human inflicted bruises in slaughter pigs are hampering animal welfare, are an infringement of the animal protection act, and are a focus of public attention. The aim of the present study was to evaluate the gross appearance of human inflicted bruises in slaughter pigs and to compare the inflammatory changes in two lesions as a basis for estimating the age of lesions in the same pig.

Pigs with human inflicted bruises slaughtered at two major slaughterhouses in Denmark from November 2013 to May 2014 were evaluated. After slaughter, the bruises were examined grossly and skin and underlying muscle tissue from two similar but separate bruises (a and b) on each pig were sampled for histology.

**Results:**

Skin and muscle tissue from 101 slaughter pigs were subjected to gross evaluation. Eighty-one of these were also subjected to histological evaluation. Most frequently (51 out of 101 pigs, 50 %), bruises had a tram-line pattern due to blunt trauma inflicted with long objects such as sticks. Other bruises reflected the use of tattoo-hammers, plastic paddles, double U profiles and chains. Histological evaluation of two bruises from a pig with multiple lesions was found insufficient to assess the overall age of the lesions as substantial variation in the inflammatory response between bruises was present.

**Conclusions:**

Grossly, the pattern of bruises often reflected the shape of the object used for inflicting the lesions. When determining the age of multiple bruises on a pig more than two lesions should be evaluated histologically.

**Electronic supplementary material:**

The online version of this article (doi:10.1186/s12917-016-0869-3) contains supplementary material, which is available to authorized users.

## Background

During recent years, human inflicted bruises in pigs have received increased attention [1–7]. Estimation of the age of such bruises is crucial in order to determine in whose custody the pig was when the lesions were inflicted [2]. The age determination of the lesions is based on a histological evaluation of skin and underlying muscle tissue [2]. Histological evaluation of skin and underlying muscle tissue is able to determine if bruises are 1 to 3 h or 4 to 10 h old based on experimental bruises in pigs [4]. Estimation of the age of bruises was primarily based on the infiltration of neutrophils in the subcutaneous tissue and of macrophages in the underlying muscle tissue.

At gross evaluation, human inflicted bruises on slaughter pigs are defined by being multiple, having a uniform pattern often identifying the object used to inflict, and are always placed on the back or upper sides of the animals [2]. Moreover, based on histological evaluation, more than 90 % of bruises on slaughter pigs are assumed to be inflicted in less than 8 h prior to slaughter, i.e., when the pigs are managed around transport to slaughter [2]. Therefore, multiple bruises on a slaughter pig are most likely of the same age (applied during a short period, i.e., minutes); and an overall assessment of the age in relation to the time of slaughter is stated [2]. However, a comparison of the inflammatory changes between individual bruises on the same animal has not previously been done, and the impact on the age determination is unknown.

As part of a study requested by the Danish Veterinary and Food Administration [1] skin and muscle tissue from human inflicted bruises on Danish slaughter pigs were sampled prospectively at two major slaughterhouses. The aim of the present study was to evaluate the gross appearance of bruises in slaughter pigs. Moreover, the inflammatory changes and the estimated age of each of two bruises sampled from pigs with multiple bruises were also compared.

## Methods

**Fig. 1:**
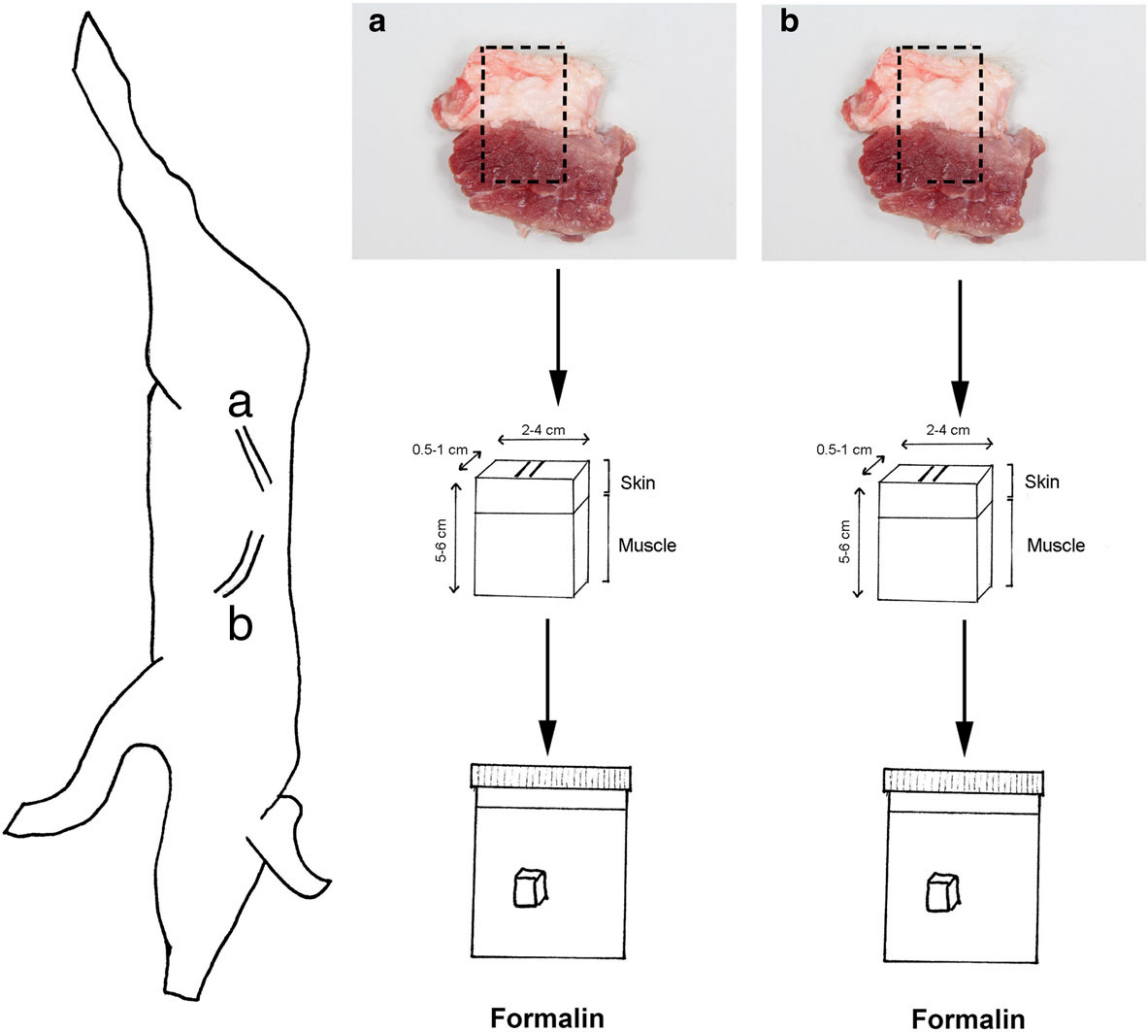
Drawing illustrating a carcass of a slaughter pig with multiple bruises. Two bruises are marked (**a** and **b**). Skin and underlying muscle tissue from bruises **a** and **b** were sampled at the slaughter houses and immersion-fixed in 10 % buffered formalin

Routine monitoring of human inflicted bruises occur at the slaughter lines in all abattoirs in Denmark. All pigs detected with human inflicted bruises slaughtered at two major slaughterhouses in Denmark from November 2013 to May 2014 were included. All pigs were slaughter pigs, i.e., around 5 to 6 months old and had a mean carcass weight of 83.25 ± 6.58 (SD) kg. The animals were all transported directly from farms to the slaughter houses within an average transportation time of 2.13 ± 3.33 (SD) h. After arrival, the animals were herded together by the use of noise making plastic paddles and were slaughtered within 3.03 ± 3.44 h (SD). After slaughter, all animals were closely examined for the presence of human inflicted bruises. Bruises are defined as being inflicted by humans if they are multiple, having a uniform pattern often reflecting the object used for infliction and are localized on the back or upper sides of the slaughter pigs [2]. After slaughter, skin and underlying muscle tissue from two separate bruises (a and b) on each pig were sampled by veterinarians employed at the slaughterhouses and immersion-fixed in 10 % neutral buffered formalin for at least 5 days (Fig. 1). The remaining skin was stored at -18 °C before being submitted for gross evaluation at the University of Copenhagen.

### Pathological evaluation

At gross evaluation, the pattern and dimensions of bruises were registered. For histology, a single tissue section of each of the immersion-fixed skin and muscle tissues (a and b) were cut at 4-5 μm and stained with hematoxylin and eosin [8]. Dermis, subcutis and muscle tissue were assessed for hemorrhage, hyper-leukocytosis (capillary pavement of leukocytes), and infiltration of neutrophils and macrophages. Hemorrhage and hyper-leukocytosis were recorded as present or absent in all tissue layers. In the dermis, infiltration of neutrophils and macrophages was registered as present or absent. In the subcutis and muscle tissue, infiltration of neutrophils and macrophages was scored on a semi-quantitative scale: 0) absence of neutrophils or macrophages, 1) 1-10 neutrophils or macrophages, 2) 11-30 neutrophils or macrophages, 3) >30 neutrophils or macrophages. The scoring was carried out by using a 40x objective in the area with the highest density of neutrophils and macrophages. In the muscle tissue, the percentile area of necrotic muscle fibers was scored according to the following scale by using a 10x objective: 0) no necrosis: absence of necrotic muscle fibers, 1) minor necrosis: <12.5 %, 2) moderate necrosis: 12.5-50 %, 3) severe necrosis: >50 %. Moreover, in the muscle tissue, the localization of neutrophils and macrophages was recorded as predominantly being present in the interstitial spaces (>50 % of the leukocytes) or as intracellular infiltrations (>50 % of the leukocytes) in the necrotic muscle fibers.

### Age estimation

The age of a bruise was determined as a time interval based on the infiltration of neutrophils and macrophages in the subcutis and muscle tissue, respectively, according to the results from experimental porcine bruises [4]. This was done by combining the age interval given by the neutrophil score together with the age interval given by the macrophage score (Table 1). In case of overlap between the age intervals, the two intervals were combined so that the narrowest age interval was obtained for each bruise. If the age interval obtained by the neutrophil score and the macrophage score did not overlap, the highest score (based on infiltration of either neutrophils or macrophages) was used to determine the age of the bruise. A score of zero was considered as inconclusive.

**Table 1:** Estimation of the age of bruises localized on the back or upper side of slaughter pigs

Cell type and tissue	Score	Bruise age
Neutrophils, subcutis	1	1 to 3 h
Neutrophils, subcutis	2	1 to 8 h
Neutrophils, subcutis	3	4 to 10 h
Macrophages, muscle	1	2 to 9 h
Macrophages, muscle	2	2 to 10 h
Macrophages, muscle	3	4 to 10 h

The age of bruises was based on the degree of infiltration by neutrophils and macrophages (cell type) in the subcutis and the muscle tissue, respectively [4]. The age of a bruise was stated by combining the age intervals given by the neutrophil score and the macrophage score

Based on the age interval, bruises were assigned to one of the following four categories: 1) inconclusive (neutrophils and macrophages were absent), 2) <4 h, 3) >4 h, and 4) age intervals overlapping 4 h (e.g., 1 to 8 h, 2 to 10 h).

### Statistics

Agreement between observations in bruises a and b was determined for each of the histological parameters and for the age of the bruises (categories 1 to 4) by calculating Cohen’s kappa using the fmsb function [9] in R version 3.2.2 [10].

## Results

Skin from 101 slaughter pigs was submitted for gross evaluation, and seven patterns of bruises were recognized either occurring alone or in combination (Table 2).

**Table 2:** The pattern of bruises reflecting the object used for infliction on 101 slaughter pigs

Pattern	Number of pigs (*n* = 101)	
Tramline	51	(50 %)
Tattoo-hammer	21	(21 %)
Paddle	8	(8 %)
Double U profile	6	(6 %)
Circle	3	(3 %)
Chain	2	(2 %)
Other	10	(10 %)

Six patterns of bruises were recognized. In 13 % of pigs the object used to inflict bruises could not be identified

**Fig. 2:**
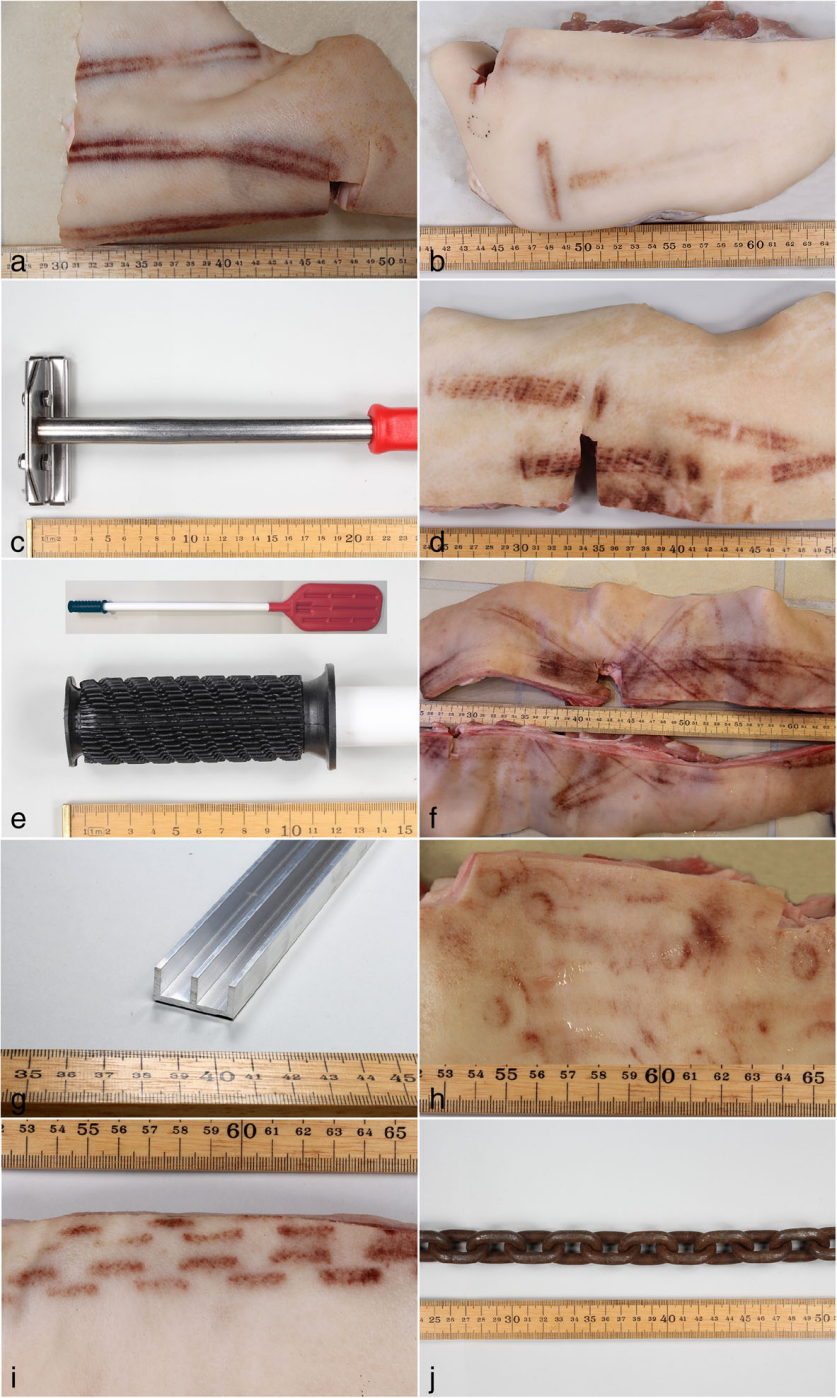
The patterns of bruises on pig skin and objects clearly used to inflict lesions. **a** Bruises with a tramline pattern compatible of being inflicted with a stick. **b** Bruises consistent with being inflicted by the back of the head of a tattoo-hammer (see **c**). **c** The back of a tattoo-hammer. **d** Bruises reflecting the handle of a plastic paddle (see **e**). **e** Handle of a plastic paddle and the entire plastic paddle (inset). **f** Bruises inflicted by a double U profile (see **g**). **g** Double U profile. **h** Multiple circle formed bruises consisting of a small circle of hemorrhage within a larger circle. The object used to inflict these bruises is unknown. **i** Bruises reflecting strikes using a chain (see **j**). **j** Chain. Ruler on figures is in cm

Tramline bruises: Bruises with a tramline pattern were characterized by two longitudinal, parallel lines of hemorrhage (width from 0.1 to 1.2 cm) separated by apparently normal skin (width from 0.2 to 3 cm). This pattern was compatible of being inflicted with a stick and was present on 51 pigs (Table 2 and Fig. 2a).

Tattoo-hammer bruises: Uniform bruises consistent with being inflicted by the back of the head of a tattoo-hammer were found on 21 pigs (Table 2). This type of pattern consisted of a short (4.5 to 7 cm) tramline pattern or a confluent accumulation of hemorrhage (Fig. 2b). On 3 out of the 21 pigs, a second hemorrhage was seen perpendicular to the first hemorrhage, i.e., compatible of being caused by the shaft of the tattoo-hammer (Figs. 2b and c).

Paddle formed bruises: Bruises reflecting the handle of a plastic paddle (“pig-paddle”) were seen on 8 pigs. The bruises appeared as oblique rows of parallel hemorrhages similar to the shape of the handle of a plastic paddle, which is a handling device normally used when herding pigs (Figs. 2d and e). Sometimes, bruises with a tramline pattern were present in proximity to the handle shaped bruises consistent with the shaft of the paddle.

Double U profile bruises: Bruises inflicted by double U profiles were present on 6 pigs (Table 2 and Figs. 2f and g). The bruises consisted of three parallel lines of hemorrhages (width from 0.1 to1.5 cm) separated by apparently normal skin (width from 0.3 to 0.8 cm).

Circle formed bruises: On 3 pigs, multiple circular hemorrhages were seen (Table 2 and Fig. 2h). The bruises consisted of a small circle of hemorrhage (diameter from 0.6 to 1 cm) within a larger circle (diameter from 2.3 to 2.5 cm). The small circles were occasionally seen as a confluent hemorrhage, and both small and large circles were sometimes open, i.e., not complete. The tool used for applying the circle formed bruises is unknown.

Chain shaped bruises: Bruises reflecting strikes using a chain were seen on 2 pigs (Table 2 and Figs. 2i and j). This type of bruise consisted of up to four rows of parallel hemorrhages with a length from 1.4 to 2.3 cm and a width from 0.2 to 0.5 cm.

Other bruises: On 10 pigs, bruises with other patterns were present (Table 2). The tools used to inflict these bruises could not be identified, and the shape of the lesions differed.

In total, skin and muscle tissue from 81 of the pigs (i.e., bruises a and b) were available for histological evaluation. From the remaining 20 pigs, the tissues were excluded from histological evaluation due to two reasons: missing samples (*n* = 15) and putrefaction of samples due to an insufficient volume of formalin (*n* = 5).

Cohen’s kappa value, *p*-value and interpretation of agreement for each of the histological parameters are presented in Table 3. A fair agreement (lowest level) was found between the age estimation of bruise a and bruise b (κ = 0.24, 95 % confidence interval = [0.08 to 0.40], *p* = 0.0008). The distribution of the age is shown in Table 4. In 48 % of pigs, bruise a and bruise b were estimated to the same age category (Table 5).

**Table 3:** Diagnostic agreement (estimated as Cohen’s kappa) between two samples (a and b) of bruises from each of 81 slaughter pigs with multiple bruises

Tissue layer	Variable	Kappa	Lower95	Upper95	Level of agreement	*P*-value
Dermis	Hyper-leukocytosis	0.58	0.29	0.88	Moderate	0.0037
	Leukocytes	0.43	0.23	0.64	Moderate	0.0002
	Hemorrhage	0.29	-0.18	0.76	Not significant	0.1508
Subcutis	Neutrophils	0.30	0.14	0.46	Fair	0.0000
	Hyper-leukocytosis	0.29	-0.18	0.76	Not significant	0.5000
	Macrophages	0.52	0.23	0.80	Moderate	0.0034
	Hemorrhage	-0.04	-0.77	0.70	Not significant	0.5395
Muscle	Necrosis	0.14	-0.19	0.48	Not significant	0.2146
	Neutrophils	0.32	0.12	0.53	Fair	0.0015
	Macrophages	0.17	-0.06	0.40	Not significant	0.0806
	Leukocytes in the interstitial space	0.43	0.15	0.71	Moderate	0.0077
	Leukocytes present intramuscularly	0.40	0.18	0.62	Moderate	0.0008
	Hyper-leukocytosis	0.00	-1.95	1.95	Not significant	0.5000
	Hemorrhage	0.34	0.13	0.54	Fair	0.0012

Limits of 95 % confidence interval not including zero and a *P*-value for kappa below 0.05 means there is some level of agreement between the test results of tissue samples a and b within the same pig. The level of agreement (fair, moderate) depends on the kappa value

The agreement was based on histological variables within the dermis, subcutis and underlying muscle tissue of bruise a and bruise b

**Table 4:** The pattern of the estimated age of two bruises (a and b) on each of 81 slaughter pigs with multiple bruises

Age	Bruise a		Bruise b	
Inconclusive	5	(6 %)	7	(9 %)
<4 h	30	(37 %)	36	(44 %)
>4 h	15	(19 %)	13	(16 %)
Overlapping 4 h	31	(38 %)	25	(31 %)

The number and percentage of bruises in each of the four age categories are presented

**Table 5:** Agreement and difference in the estimated age of bruise a and bruise b on each of 81 pigs

		Bruise a			
		Inconclusive	<4 h	>4 h	Overlapping 4 h
Bruise b	Inconclusive	1	3	0	3
	<4 h	1	20	3	12
	>4 h	0	3	6	4
	Overlapping 4 h	3	4	6	12

## Discussion

In approximately half of the slaughter pigs, the bruises had a tram-line pattern similar to the pattern previously described in pigs, cattle and humans beaten with sticks such as broom handles or other wood and metal rods [2, 4, 6, 11–14]. Moreover, in a study from Brazil, bruises in pigs with a rectangular shape were associated to be inflicted by sticks and other solid handling devices when herding the pigs [5]. The pattern of some bruises clearly revealed the equipment used to inflict the bruises, e.g., the back of a tattoo-hammer, the handle of a plastic paddle or a chain, which are all tools or parts of equipment that are normally present in a pig production environment. Tattoo-hammers are normally used for applying an identification number on the pigs at the farm, while plastic paddles are used for herding pigs together on farms and at slaughter houses.

Histologically, the degree of inflammatory changes and the age assessment varied between bruise a and bruise b as none of the kappa values exceeded 0.6. The variation in the inflammatory response between the two bruises is in accordance with human cases, in which bruises with and without inflammatory changes coexisted although they were known to have been established 30 h or more before death [15]. In addition, the degree of inflammatory changes has been seen to vary between experimental bruises of the same age on the same pig [4]. In the present study, the exact age of the bruises was unknown. However, due to the uniform pattern of bruises on each pig, it is reasonable to assume that they were inflicted almost simultaneously (i.e., within minutes) at a given time before slaughter. In forensic cases of multiple bruises on a pig, all lesions are presumed to have been inflicted almost at the same time (within minutes) [2]. Therefore, one overall assessment of the age is made for all bruises based on the examination of an unspecified number of samples of skin and underlying muscle tissue [2]. However, due to the difference regarding the age estimation and the variation in the inflammatory response in bruise a and bruise b, histological evaluation of skin and muscle from two bruises is insufficient in order to determine an overall state of lesions and thereby a common age of bruises on a pig, despite being inflicted almost simultaneously.

## Conclusions

Grossly, the pattern of bruises in slaughter pigs often reflected the shape of the object used for inflicting the lesions. Most frequently, bruises had a tram-line pattern due to blunt trauma inflicted with long objects such as sticks. Histological evaluation of two bruises was insufficient to determine the age (i.e., at what time before slaughter the pigs were beaten) as substantial variation in the inflammatory response between the two bruises (a and b) was present.

## Additional file

Additional file 1: Histological evaluation of bruises. Presence/absence (1/0) of: hyper-leukocytosis, leukocytes and hemorrhage in the dermis; neutrophils, hyper-leukocytosis, macrophages and hemorrhage in the subcutis; necrosis, neutrophils, macrophages, leukocytes in the interstitial space, leukocytes present intramuscularly, hyper-leukocytosis and hemorrhage in the muscle tissue. (XLSX 17 kb)
